# Drug-associated inflammatory bowel disease: a real-world pharmacovigilance study using the FAERS and JADER databases

**DOI:** 10.3389/fimmu.2026.1873829

**Published:** 2026-07-07

**Authors:** Yuou Ying, Mengyuan Shen, Jinhan Chen, Tongfei Feng, Zejiong Li, Zhekai Ying, Dongdong Yang, Ruyi Ju, Jiannong Wu

**Affiliations:** 1 The First Affiliated Hospital of Zhejiang Chinese Medical University (Zhejiang Provincial Hospital of Chinese Medicine), Hangzhou, China; 2 Hangzhou Third People’s Hospital, Hangzhou Third Hospital Affiliated to Zhejiang Chinese Medical University, Hangzhou, China; 3 Department of Intensive Care Unit, The First Affiliated Hospital of Zhejiang Chinese Medical University (Zhejiang Provincial Hospital of Chinese Medicine), Hangzhou, China

**Keywords:** adverse events, FAERS, inflammatory bowel disease, JADER, pharmacovigilance

## Abstract

**Background:**

Although several medications have been linked to inflammatory bowel disease (IBD), the association between most drugs and IBD remains unclear. This study aimed to identify medications most frequently reported in connection with IBD by analyzing large-scale data from the U.S. Food and Drug Administration Adverse Event Reporting System (FAERS) and the Japan Adverse Drug Event Report (JADER) database.

**Methods:**

We extracted reports of drug-associated IBD adverse events from FAERS and performed external validation using JADER. Disproportionality analyses of suspected drugs were conducted using four methods: Reporting Odds Ratio (ROR), Proportional Reporting Ratio (PRR), Bayesian Confidence Propagation Neural Network (BCPNN), and Multi-Item Gamma Poisson Shrinker (MGPS). Additional analyses included time-to-onset (TTO) assessment, Weibull distribution modeling, and subgroup analyses by age, sex, and IBD subtype to evaluate signal stability and clinical characteristics.

**Results:**

The FAERS database yielded 52,395 reports of drug-associated IBD, corresponding to 50,426 patients.Females accounted for a higher proportion of reports than males, with the majority of cases occurring in individuals aged 18–64 years. The annual number of reports showed an overall upward trend. Among drugs reported at least 50 times, 17 met all four algorithmic threshold criteria and were classified as positive signals. Anti–tumor necrosis factor-alpha (anti-TNF-α) inhibitors ranked highest by reporting frequency, whereas isotretinoin demonstrated the strongest signal intensity. External validation using JADER confirmed consistent signals for certain anti-TNF-α inhibitors, anti–interleukin biologic agents, conventional immunosuppressants, and antibiotics. Time-to-onset analysis revealed that the median onset of IBD occurred within one year for most drug-associated cases. The Weibull model indicated an “early failure–type” onset pattern for the majority of positive signals.

**Conclusion:**

From a pharmacovigilance perspective, this study provides a comprehensive overview of medications associated with IBD, offering clinically relevant insights. Our findings deliver real-world evidence to support the identification and monitoring of drug-associated IBD; however, confirmation in large prospective cohort studies remains necessary.

## 1 Introduction

Inflammatory bowel disease (IBD), which includes Crohn’s disease (CD) and ulcerative colitis (UC), comprises a group of chronic inflammatory disorders of the gastrointestinal tract driven by immune dysregulation. Common symptoms include diarrhea, abdominal pain, and rectal bleeding (1). In severe cases, IBD can lead to complications such as intestinal obstruction, perforation, abscess formation, and colorectal cancer (2). According to the Global Burden of Disease Study 2021, approximately 3.8 million people worldwide are affected by IBD, with around 375,000 new diagnoses and 42,000 deaths annually (3). The global incidence of IBD is rising, particularly in newly industrialized countries (4). Given its chronic, incurable, and unpredictable course, IBD requires lifelong management, imposing substantial health and economic burdens on both patients and healthcare systems.

The pathogenesis of IBD is multifactorial, involving complex interactions among genetic, immunological, microbial, and environmental factors (5). Adverse drug reactions, especially those affecting the gastrointestinal tract, are frequently encountered in clinical practice. A growing body of evidence suggests that certain medications may trigger new-onset IBD. For instance, non-steroidal anti-inflammatory drugs (NSAIDs) and antibiotics have been associated with incident IBD despite their well-established therapeutic benefits (6). More recently, a paradoxical phenomenon has emerged: IBD treatment agents, including anti–tumor necrosis factor-alpha (anti-TNF-α) biologics, anti–interleukin (anti-IL) biologics, and immunomodulators, have been linked not only to disease exacerbation but also to *de novo* IBD in some patients. Although the mechanisms underlying these effects may differ, such occurrences are not uncommon (7). Therefore, vigilant monitoring for drug-associated IBD and timely identification of associated risks are essential to prevent this complication.

The U.S. Food and Drug Administration (FDA) Adverse Event Reporting System (FAERS) and the Japanese Adverse Drug Event Report (JADER) database, maintained by Japan’s Pharmaceuticals and Medical Devices Agency (PMDA), are two well-established spontaneous reporting systems. FAERS aggregates a large volume of adverse event reports primarily from the United States, while JADER compiles similar data from Japan. With more than 20 million records, FAERS is the largest global database of its kind and provides broad insights into drug safety across diverse patient populations (8). Although smaller and focused on the Japanese population, JADER offers unique value in evaluating drug-related outcomes in Asian patients, particularly for domestically used medications. Despite differences in data structure and reporting practices, the complementary geographic and demographic coverage of FAERS and JADER enables cross-validation and strengthens the reliability of signal detection.

Leveraging these resources, this study systematically analyzes adverse event reports related to drug-associated IBD in FAERS from the first quarter of 2004 through the second quarter of 2025. The JADER database is used for external validation to confirm the robustness of the findings. Identifying medications associated with IBD and implementing appropriate preventive strategies are critical steps toward reducing the burden of drug-associated IBD. In summary, this study aims to uncover drugs linked to IBD and provide clinically relevant insights into their comparative safety profiles to support improved therapeutic decision-making and drug management.

## 2 Methods

### 2.1 Data sources and collection

This pharmacovigilance analysis used data from the FAERS and JADER databases. We extracted reports of drug-associated IBD from FAERS covering the period from the first quarter of 2004 to the second quarter of 2025. The FAERS data consist of seven linked datasets: DEMO (Demographics), DRUG (Medication), REAC (Adverse Events), OUTC (Outcomes), RPSR (Reporter), THER (Therapy Duration), and INDI (Indication), which are connected through the identifiers PRIMARYID and CASEID.

Similarly, we retrieved reports of drug-associated IBD from JADER spanning January 2004 to November 2025. JADER includes four datasets: DEMO, DRUG, REAC, and HIST. The DEMO file contains basic patient information such as sex, age, and weight. The DRUG file provides medication details, including generic names, routes of administration, and start and end dates of drug use. The REAC file records adverse event names, outcomes, and occurrence dates. The HIST file documents the patient’s primary underlying diseases.

In both the FAERS and JADER databases, all adverse events (AEs) were coded using Preferred Terms (PT) from the Medical Dictionary for Regulatory Activities (MedDRA), Version 27.0, enabling hierarchical classification into High-Level Terms (HLT), High-Level Group Terms (HLGT), and System Organ Classes (SOC).Both FAERS and JADER are publicly accessible pharmacovigilance databases containing fully anonymized and deidentified patient records. Accordingly, this study was exempt from ethical review and did not require informed consent.

### 2.2 Data pre-processing and extraction

In accordance with FDA guidelines, duplicate reports were removed by matching the variables PRIMARYID, CASEID, and FDA_DT from the DEMO table. For records sharing the same CASEID, the one with the most recent FDA_DT was retained; among those with identical CASEID and FDA_DT values, the report with the largest PRIMARYID was kept. We filtered the “drugname” and “prod_ai” fields in the DRUG table, restricted the “role_cod” field to “PS” (primary suspect), and limited the “PT” field to “IBD,” “ulcerative colitis,” and “Crohn’s disease.” Similarly, in the JADER database, duplicate rows were removed from the REAC table by matching the variables PRIMARYID, CASEID, and EVENT_DT. If all three matched, the row was considered a duplicate and excluded.The identification of “inflammatory bowel disease,” “ulcerative colitis,” and “Crohn’s disease” in the JADER database followed the same rules as those applied to FAERS, with the “role_cod” field restricted to the Japanese qualifier meaning the same as “PS”.Additionally, because the same condition may be recorded as both an indication and an adverse event in FAERS and JADER, we excluded IBD and its subtypes from the list of indications during case retrieval to avoid misclassifying therapeutic use as an adverse reaction. Furthermore, drugs approved by the FDA exclusively for IBD treatment were excluded. This stringent criterion aimed to minimize indication-related confounding. Nevertheless, we recognize that distinguishing disease-related symptoms from true drug-associated AEs remains inherently challenging in spontaneous reporting systems due to the lack of detailed clinical context.

### 2.3 Data mining and statistical analysis

This study used disproportionality analysis as the primary signal detection approach, supported by four statistical algorithms: Reporting Odds Ratio (ROR), Proportional Reporting Ratio (PRR), Information Component (IC), and Empirical Bayes Geometric Mean (EBGM) (see **Supplementary Table 1** for specific formulas). A drug–AE combination was considered a positive signal only if it simultaneously met the predefined positivity criteria for all four methods. Specifically, for ROR, a signal required at least three reports and a lower 95% confidence interval (CI) greater than 1; for PRR, it required at least three reports, a PRR value of 2 or higher, and a chi-squared (χ²) statistic of at least 4; for IC, the lower bound of the 95% CI (denoted IC_025_) had to exceed 0; and for EBGM, the lower 95% CI (EBGM_05_) needed to be greater than 2. The p-values obtained from Fisher’s exact test were adjusted using the Bonferroni correction.Time to onset was defined as the interval between drug administration and the reported onset of IBD. Records with missing, invalid, or negative time-to-onset values were excluded, and the median time (in days) along with its interquartile range (IQR) was calculated. The Weibull distribution model was employed to dynamically analyze the temporal evolution of AE onset, characterized by two core parameters: scale (α) and shape (β). This model facilitated a prognostic evaluation of how the risk of IBD changes over time following drug exposure.Subgroup analyses stratified by age, sex, and disease subtype (CD versus UC) were conducted to assess the robustness of the detected medication–IBD signals across distinct patient populations. External validation was performed using the Japanese JADER database. The same disproportionality analysis algorithms and positivity thresholds were applied to the JADER database as those used for FAERS.All statistical analyses were carried out in SAS 9.4, and data visualization was generated using R 4.0.2 and GraphPad Prism 10.6.1.

## 3 Results

### 3.1 Descriptive statistics

**Figure 1:**
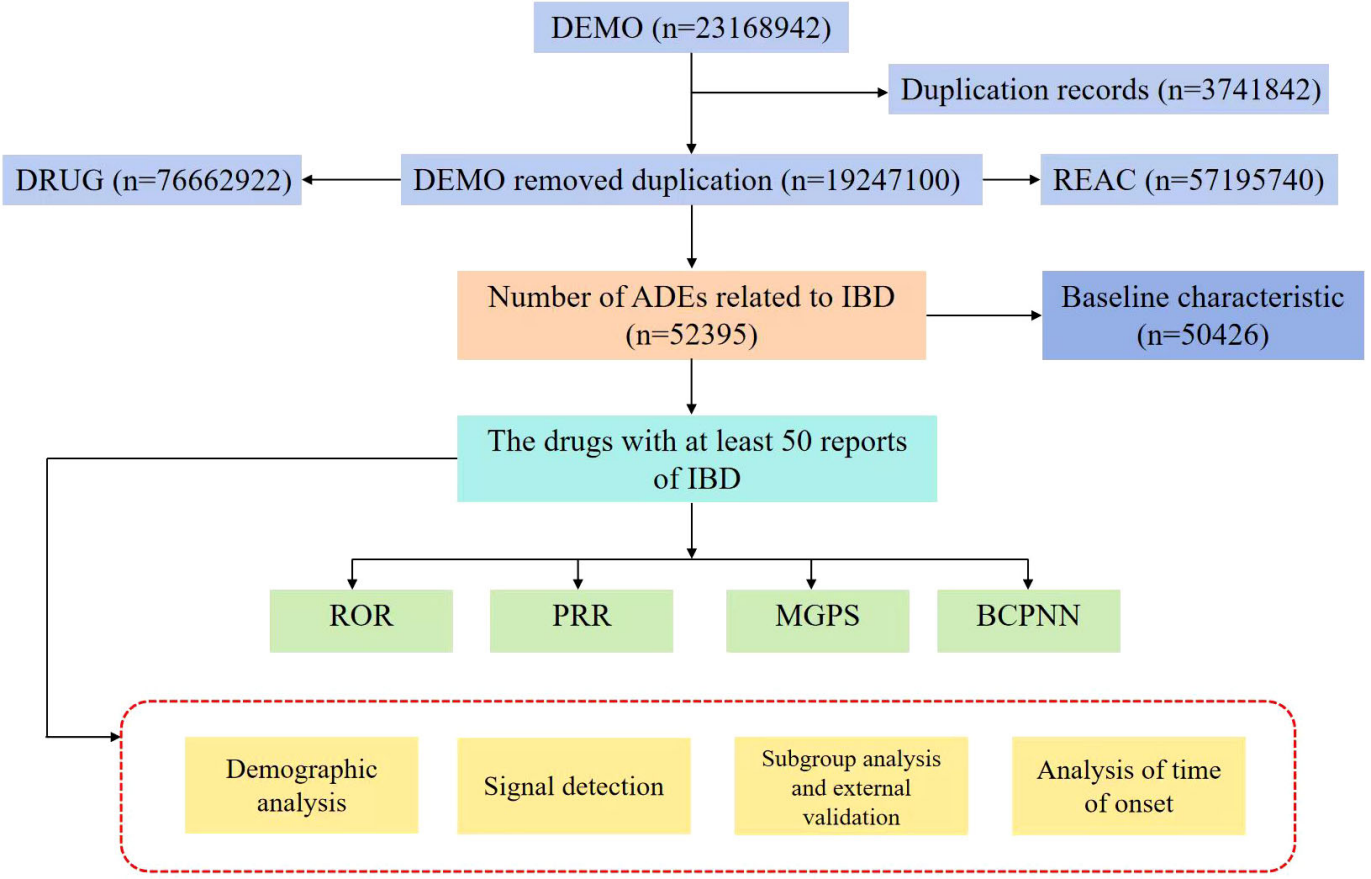
The flow diagram of the study. DEMO, demographic and administrative information; DRUG, drug information; REAC, coded AEs.

**Figure 2:**
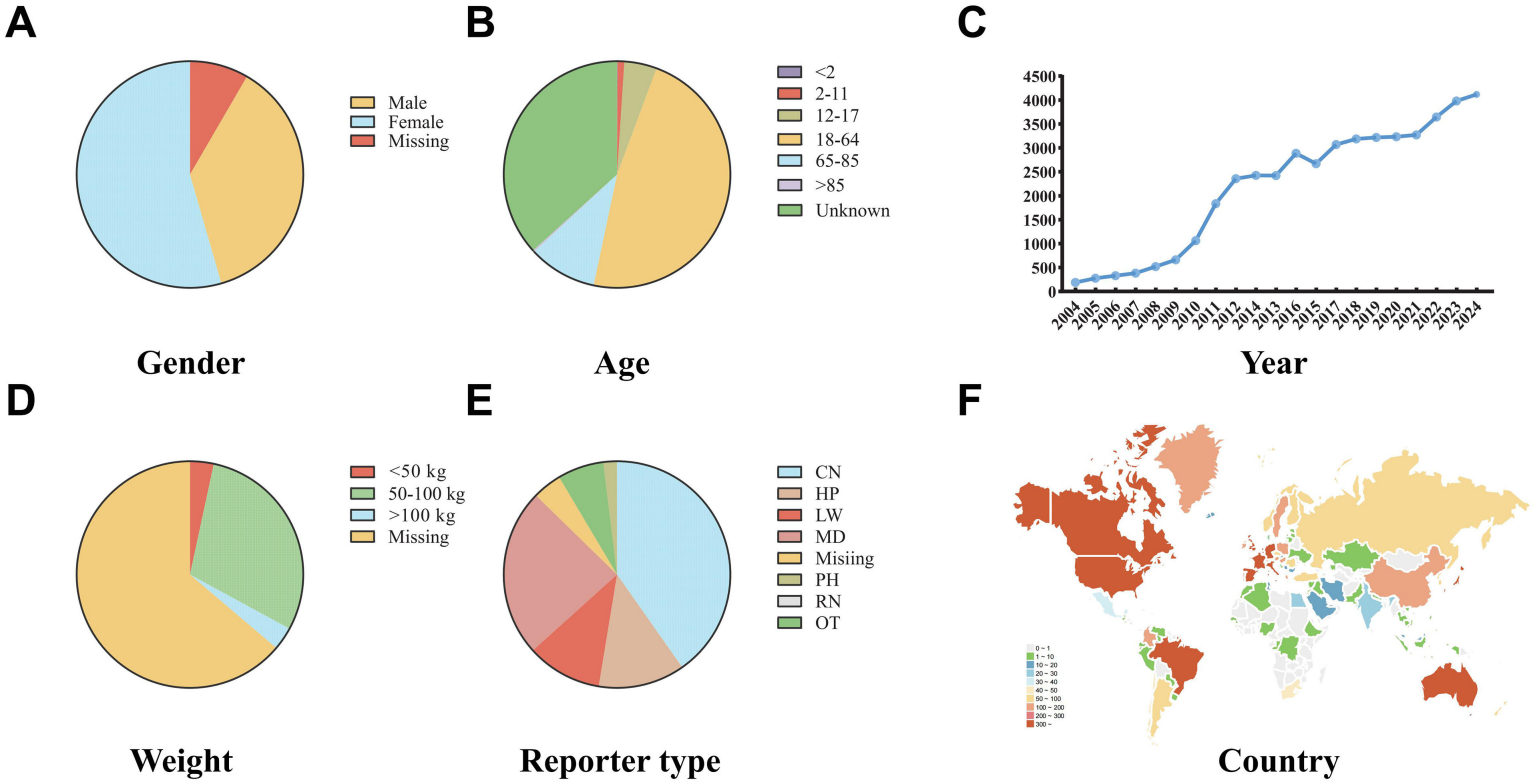
The demographic characteristics distribution of drug-associated IBD patients in the FAERS database. **(A)** Distribution of gender; **(B)** Distribution of age; **(C)** Distribution of reporting years; **(D)** Distribution of weight; **(E)** Distribution of reporting type; **(F)** Distribution of countries.

A comprehensive search of the FAERS database yielded 52,395 reports related to drug-associated IBD, involving 50,426 patients. (see **Figure 1**, **Supplementary Table 2**). Females accounted for a larger share of reports than males (n = 27,467 [54.5%] vs. n = 18,731 [37.1%]) (**Figure 2A**). The majority of cases occurred in individuals aged 18–64 years (n = 24,025, 47.6%) (**Figure 2B**). Among those with recorded weight, the 50–100 kg range was most common (n = 14,899, 29.5%) (**Figure 2D**). Consumers constituted the largest reporting group (n = 20,295, 40.2%), followed by physicians (n = 12,085, 24.0%) and other health professionals (n = 6,228, 12.4%) (**Figure 2E**). The annual number of reported cases showed a steady upward trend over time (**Figure 2C**). Geographically, North America contributed the greatest proportion of reports, led by the United States (n = 23,358, 46.3%) and Canada (n = 10,731, 21.3%) (**Figure 2F**). Regarding clinical outcomes, 37.7% of cases resulted in hospitalization, while the mortality rate was 2.8%, indicating that hospitalization was considerably more frequent than death.

### 3.2 Disproportionality analysis in FAERS

**Figure 3:**
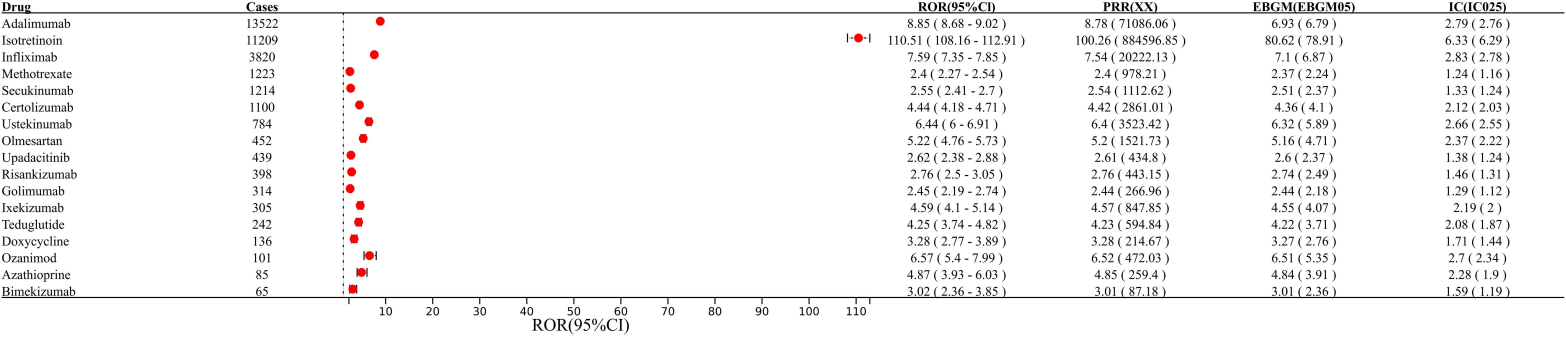
Signal detection results for drugs with at least 50 reports in the FAERS database. ROR, the reporting odds ratio; PRR, the proportional reporting ratio; EBGM, the empirical Bayes geometric mean; EBGM05, the lower limit of 95% CI, of EBGM; IC, the information component; IC025, the lower limit of 95% CI, of the IC.

To evaluate associations between medications and IBD, we conducted disproportionality analyses using ROR, PRR, BCPNN, and MGPS methods. Among drugs with at least 50 reports, 17 generated positive signals across all four algorithms (**Supplementary Table 3**). Adalimumab had the highest report count (13,522 cases), followed by isotretinoin (11,209 cases) and infliximab (3,820 cases). In terms of signal strength, isotretinoin exhibited the strongest association, with an ROR of 110.51, followed by adalimumab (ROR = 8.85), infliximab (ROR = 7.59), ozanimod (ROR = 6.57), and ustekinumab (ROR = 6.44), which together ranked as the top five signals. Based on mechanism of action, these agents fall into several categories: anti-TNF-α inhibitors, anti-interleukin biologics, conventional immunosuppressants, and individual drugs with diverse mechanisms, including doxycycline, olmesartan, and teduglutide (**Figure 3**).

### 3.3 Subgroup stratification analysis

To assess robustness, we performed predefined stratified subgroup analyses (**Supplementary Table 4**). Age-stratified disproportionality analysis revealed notable differences in drug–IBD signals between age groups. Using ROR as the metric, most drugs showed higher associations in patients aged ≥65 years compared with those <65 years. In contrast, isotretinoin and ozanimod exhibited significantly stronger signals in the younger subgroup (<65 years). A positive signal for pantoprazole and IBD was detected exclusively in patients aged ≥65 years.Gender-based stratification indicated that isotretinoin, ozanimod, upadacitinib, and most anti-TNF-α agents had higher RORs in males, whereas doxycycline generated a positive signal only in females. Additionally, pantoprazole, pembrolizumab, prednisone, and prednisolone showed positive signals specifically in the female subgroup. When stratified by disease subtype, isotretinoin demonstrated a significantly higher ROR in UC than in CD. Positive signals for pantoprazole, hydrocortisone, and pembrolizumab were observed only in the UC subgroup. Conversely, RORs for most anti-TNF-α drugs were greater in CD patients than in those with UC.

### 3.4 External validation

**Figure 4:**
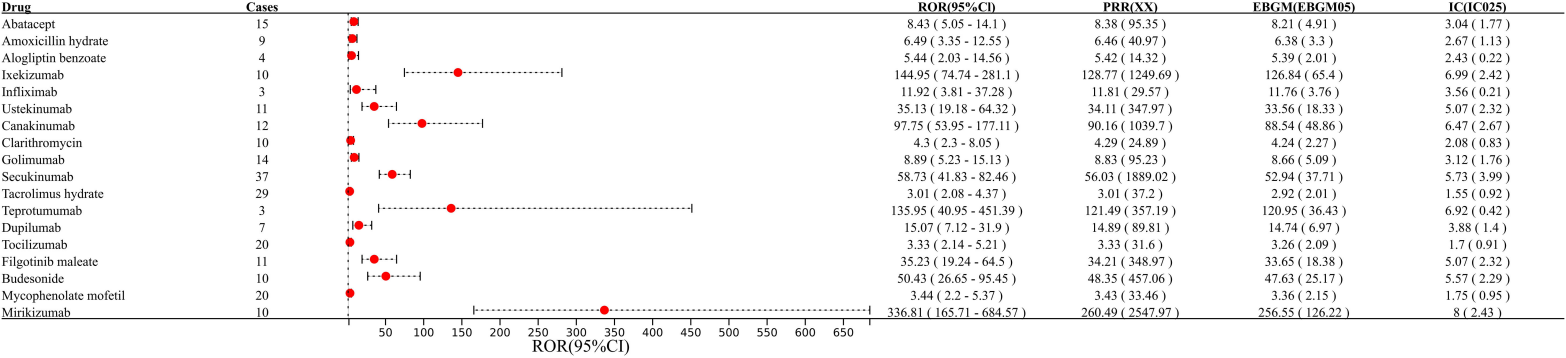
Signal detection results for drugs with at least 3 reports in the JADER database. ROR, the reporting odds ratio; PRR, the proportional reporting ratio; EBGM, the empirical Bayes geometric mean; EBGM_05_, the lower limit of 95% CI, of EBGM; IC, the information component; IC_025_, the lower limit of 95% CI, of the IC.

To mitigate potential bias in FAERS arising from incomplete adverse drug reaction reports and uncertain causality, we extracted IBD-related ADR reports from the Japanese JADER database covering 2004 onward (see **Supplementary Table 5**) and conducted signal detection for drugs reported at least three times (**Figure 4**). Analysis of JADER data showed that the primary drug classes associated with IBD included anti-TNF-α inhibitors, anti-leukocyte biotherapeutic agents, and conventional immunosuppressants.

Further cross-database verification confirmed that most drug-class signals detected in FAERS were replicated in JADER. These included TNF-α inhibitors, anti-leukocyte biotherapeutics, conventional immunosuppressive agents, and antibiotics. Collectively, this external validation supports the reliability of the disproportionality signals observed in the FAERS database.

### 3.5 Median time to onset and onset model

**Figure 5:**
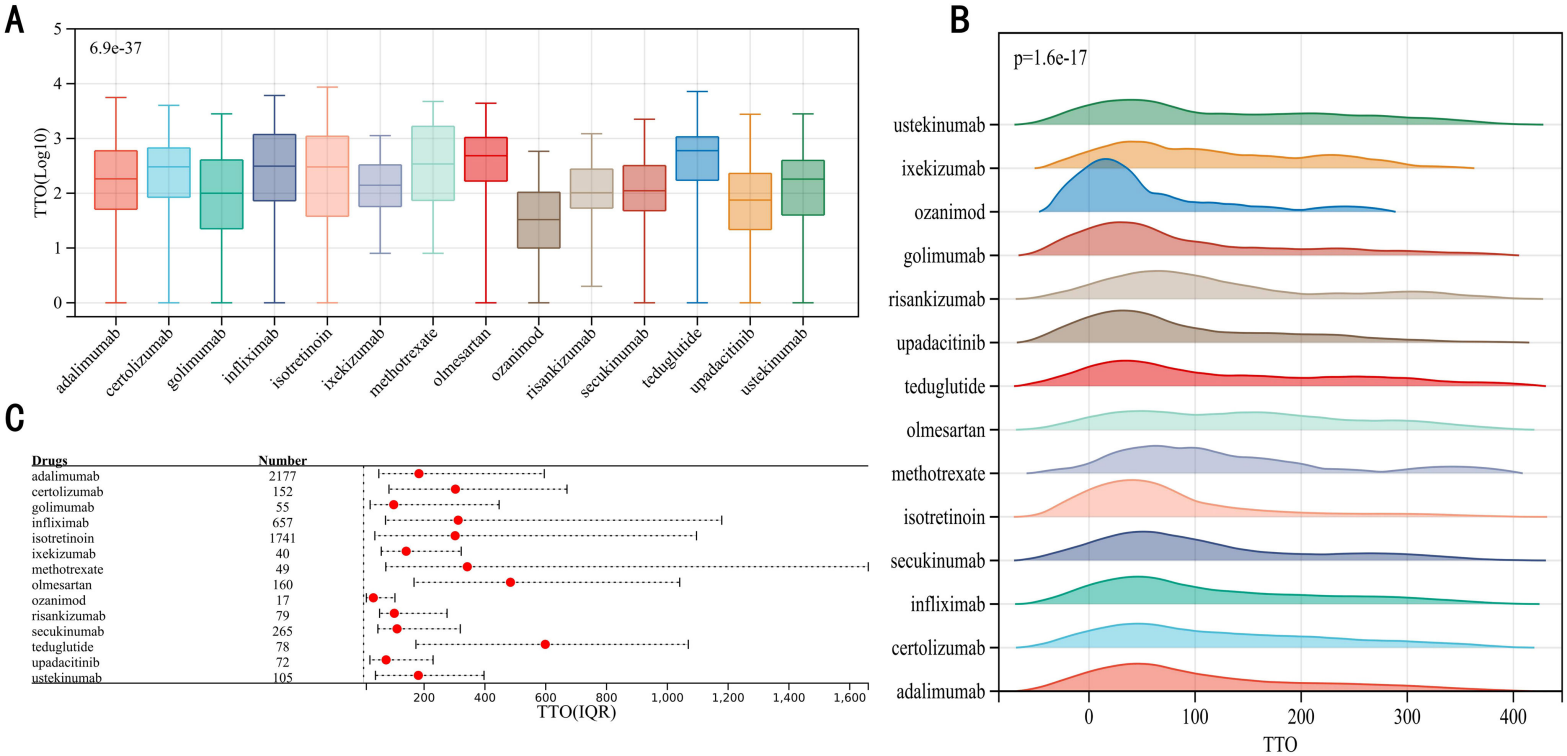
Time-to-onset analysis of IBD for different drugs. **(A)** Visualized box plot of onset time of included drugs; **(B)** Visualized mountain plot of onset time of included drugs; **(C)** The Forest Plot of the onset time of drug-associated IBD.

To characterize the time-to-onset (TTO) profile of drug-associated IBD, we analyzed positive signals from the FAERS database. A total of 5,673 IBD cases with complete TTO data were included, representing 16.02% of the cohort; the remaining 83.98% of reports were excluded due to missing medication start dates, missing adverse event dates, or non-positive TTO values (i.e., zero or negative). Because individual case reports may involve multiple suspected drugs with documented start dates, TTO was evaluated at the report–drug pair level. Notably, azathioprine (8 cases), bimekizumab (8 cases), and doxycycline (10 cases) were excluded from this analysis owing to insufficient sample sizes, which would compromise result reliability. Most drugs associated with IBD exhibited a median TTO within one year. Specifically, ozanimod had a median TTO of 33 days (IQR: 10–104 days) and upadacitinib 75 days (IQR: 21.75–229.5 days), indicating relatively early onset. In contrast, olmesartan and teduglutide showed markedly longer median TTOs, both exceeding 400 days. According to the Weibull distribution model, 11 of the 14 included drugs (78.57%) followed an early failure pattern. The remaining three drugs (21.43%), ixekizumab, risankizumab, and teduglutide, exhibited a random failure pattern (**Table 1**, **Figure 5**).

**Table 1:** Time-to-onset analysis of IBD for different drugs.

Drug	n	TTO (IQR)	Shape parameter: β (95% CI)	Scale parameter: α (95% CI)	Failure type
adalimumab	2177	183(51-595.25)	0.69 (0.67, 0.71)	346.57 (324.30, 368.84)	Early failure
certolizumab	152	303(84.5-670)	0.75 (0.66, 0.85)	476.66 (370.63, 582.68)	Early failure
golimumab	55	100(22.5-446.75)	0.57 (0.45, 0.68)	244.93 (124.36, 365.50)	Early failure
infliximab	657	312(73-1179)	0.67 (0.63, 0.71)	639.95 (590.75, 688.58)	Early failure
isotretinoin	1741	302(38-1096)	0.60 (0.58, 0.63)	569.95 (523.04, 616.86)	Early failure
ixekizumab	40	141(59-322)	0.86 (0.65, 1.07)	245.04 (151.83, 338.25)	Random failure
methotrexate	49	342(74-1661)	0.72 (0.56, 0.88)	727.72 (428.85, 1026.59)	Early failure
olmesartan	160	484(166.75-1040.75)	0.86 (0.75, 0.96)	728.50 (589.94, 867.07)	Early failure
ozanimod	17	33(10-104)	0.60 (0.38, 0.82)	66.76 (10.99, 122.53)	Early failure
risankizumab	79	102(53.5-275.5)	0.95 (0.79, 1.11)	178.50 (134.68, 222.32)	Random failure
secukinumab	265	111(48-319)	0.76 (0.69, 0.83)	218.92 (182.37, 255.46)	Early failure
teduglutide	78	598.5(173-1069)	0.86 (0.71, 1.01)	747.87 (545.51, 950.23)	Random failure
upadacitinib	72	75(21.75-229.5)	0.65 (0.53, 0.76)	159.57 (99.32, 219.81)	Early failure
ustekinumab	105	181(40-397)	0.75 (0.64, 0.86)	273.85 (200.18, 347.52)	Early failure

Based on the Weibull shape parameter (β) and its 95% confidence interval (CI), adverse event patterns are defined as follows: Early failure type: β < 1 and upper 95% CI < 1, characterized by an initial rise in risk followed by a decline. Wear-out failure type: β > 1 and lower 95% CI > 1, indicating a progressive increase in risk over time. Random failure type: β = 1 or 95% CI includes 1, reflecting a constant risk throughout treatment.

β, shape parameter; CI, confidence interval; n, number of reports with available time-to-onset; TTO, time-to-onset; α, scale parameter.

## 4 Discussion

To our knowledge, this study represents the first and largest real-world investigation of drug-associated IBD adverse events using the FAERS and JADER pharmacovigilance databases. We performed a comprehensive pharmacovigilance analysis of drug-associated IBD across both databases, including an in-depth assessment of time to onset. In total, we identified 17 drugs in FAERS and 18 in JADER that generated significantly positive disproportionality signals for drug-associated IBD.

### 4.1 Population characteristics of drug-associated inflammatory bowel disease

Our findings show that the number of reported IBD cases is greater in females than in males, consistent with prior studies (9). This disparity may stem from the influence of sex hormones on immune regulation. Estrogen exerts complex immunomodulatory effects, it can enhance certain immune responses while potentially promoting autoimmune reactions under specific conditions, thereby influencing IBD onset and progression (10). Age distribution analysis revealed the highest number of reports in the 18–65 age group. This cohort experiences greater exposure to medications due to its central role in social and healthcare activities. Notably, this range overlaps with the established peak incidence period for IBD, which typically occurs between the second and fourth decades of life (11). Geographically, the United States accounted for the largest number of reports, a pattern likely reflecting the location of the FAERS reporting system rather than true geographic variation in drug-associated IBD. Most reported outcomes were severe: 37.7% of patients required hospitalization, 2.8% died, and 47.7% experienced other serious consequences. These findings underscore the need for clinicians to remain vigilant about the potential for drug-associated IBD, particularly in patients with clinical characteristics associated with the disease.

### 4.2 Drug classes with positive signals in both FAERS and JADER

Disproportionality analyses of FAERS and JADER identified four major drug classes consistently associated with positive IBD signals: anti-TNF-α agents, anti-interleukin biologics agents, conventional immunosuppressive agents, and antibiotics.

#### 4.2.1 Anti-TNF-α inhibitors

Anti-TNF-α inhibitors target the pro-inflammatory cytokine TNF-α, thereby modulating immune signaling within inflammatory pathways. This class includes adalimumab, infliximab,certolizumab, and golimumab, which are used to treat rheumatoid arthritis, ankylosing spondylitis, psoriasis, and IBD. However, *de novo* IBD has been reported in patients with other autoimmune conditions following TNF inhibitor therapy (12). Uskudar et al. found that ankylosing spondylitis patients receiving anti-TNF treatment had a fourfold higher risk of developing new-onset IBD compared with those on other medications (13). Mechanistically, anti-TNF-α biologics not only block the pro-inflammatory actions of TNF-α but also interfere with its immunomodulatory functions, including T-cell receptor signaling, B-cell proliferation, and dendritic cell maturation, effects that may contribute to IBD emergence (14). Furthermore, TNF-α can suppress regulatory T cells, which are critical for preventing pathogenic T-cell responses against gut microbiota. In this context, TNF-α may exert a protective rather than pathogenic role (15).

#### 4.2.2 Anti-interleukin biological agents

Biologics targeting interleukins represent a rapidly evolving therapeutic frontier, with agents directed against IL-12, IL-23, IL-17, and related pathways. Ustekinumab, a fully human IgG1κ monoclonal antibody, binds the shared p40 subunit of IL-12 and IL-23, inhibiting their activity. It is widely used for psoriasis and moderate-to-severe Crohn’s disease (16). Nevertheless, some studies suggest ustekinumab may exacerbate Crohn’s disease during treatment. Although reports of IBD-related paradoxical reactions to ustekinumab remain limited and underlying mechanisms unclear, cytokine imbalance is considered the most plausible explanation (17).

Ixekizumab and secukinumab are monoclonal antibodies that neutralize IL-17A and are commonly prescribed for chronic plaque psoriasis, psoriatic arthritis, and ankylosing spondylitis. A large population-based retrospective cohort study indicated that anti–IL-17 therapy, particularly secukinumab, may be associated with an increased risk of new-onset IBD, warranting clinical vigilance (18). A phase III clinical trial also noted potential adverse events with ixekizumab, including incident IBD cases (19). Clinical experience further documents confirmed cases of new-onset IBD following treatment with either agent (20). This may reflect the dual role of IL-17A as both a pro-inflammatory and protective factor in the gut; its inhibition could disrupt mucosal homeostasis, especially in settings where the microbiome exerts strong influence (21, 22). This may reflect the dual role of IL-17A as both a pro-inflammatory and protective factor in the gut; its inhibition could disrupt mucosal homeostasis, especially in settings where the microbiome exerts strong influence (23, 24). Additionally, our study detected a positive signal between bimekizumab, a dual IL-17A/IL-17F inhibitor, and IBD. Prior research on bimekizumab for active psoriatic arthritis reported newly emerged IBD and gastrointestinal adverse reactions, though specific mechanisms remain to be elucidated (25).

#### 4.2.3 Conventional immunosuppressive agents

Azathioprine and tacrolimus are classic immunosuppressants primarily used to prevent organ transplant rejection and treat various autoimmune diseases. However, post-transplant immunomodulator use can downregulate regulatory T cells in the colonic mucosa (26). Given that regulatory T cells critically inhibit B-cell and cytotoxic T-lymphocyte activation, this downregulation may heighten susceptibility to immune-mediated colonic inflammation. Notably, multiple studies report that dual therapy with tacrolimus and mycophenolate mofetil (MMF) significantly increases the risk of IBD onset or exacerbation, whereas azathioprine tends to exert a protective effect, a finding inconsistent with our results (27, 28). Tacrolimus suppresses interleukin-2 production, thereby promoting regulatory T-lymphocyte generation; however, it also elevates the risk of intestinal dysbiosis, intestinal infections, and increased intestinal permeability. These effects expose intestinal mucosal components to the immune system, potentially driving IBD development (29). The association between MMF and IBD appears attributable to the combined actions of mycophenolic acid (MPA) and its metabolite acyl glucuronide (AcMPAG) (30). We also reidentified a positive disproportionality signal for methotrexate and IBD in the FAERS database. However, methotrexate is predominantly prescribed for immune-mediated conditions, which inherently involve immune dysregulation and elevated autoimmune comorbidity risk. Thus, the elevated ROR observed for azathioprine and methotrexate in this study likely reflects confounding by underlying disease activity rather than a direct drug effect.

#### 4.2.4 Antimicrobials

Our study shows that several broad-spectrum antibiotics are positively associated with IBD-related signals, including doxycycline, amoxicillin, and clarithromycin. A Korean population-based study demonstrated a dose-dependent increase in IBD risk with broad-spectrum antibiotic use (31). Similarly, a recent large-scale 10-year Swedish study confirmed this association, revealing a strong positive correlation between antibiotic exposure and IBD, particularly pronounced for broad-spectrum agents (32). Regarding specific antibiotic classes, a retrospective cohort study using the UK Health Improvement Network (THIN) database linked doxycycline use to an elevated risk of IBD, especially Crohn’s disease (33). Another study identified the strongest associations between IBD risk and penicillins or macrolides (34). Despite ongoing controversy, accumulating observational evidence supports a link between antibiotic use and IBD incidence. Mechanistically, doxycycline may induce gut dysbiosis, disrupt the intestinal epithelial barrier, and impair host immune tolerance, features commonly observed in IBD pathogenesis (35). Alternatively, antibiotic use might simply reflect underlying infections, which themselves could trigger IBD (36). Given the unresolved nature of current evidence, large-scale prospective studies are needed to clarify the causal relationship between antibiotic exposure and IBD development.

### 4.3 Positive signals appeared only in FAERS

Isotretinoin, a synthetic vitamin A derivative, is widely used to treat severe acne. Case reports and clinical studies suggest an association between isotretinoin and IBD (37, 38). Meta-analyses further indicate that isotretinoin use for over one year may be linked to an increased risk of IBD (39). Mechanistically, isotretinoin inhibits intestinal mucosal cell proliferation, a process critical for maintaining gut homeostasis alongside rapid epithelial turnover and T-cell activation. Moreover, isotretinoin-activated T cells express α4β7 integrin and CCR9 receptors, which play pivotal roles in initiating and sustaining gastrointestinal inflammation (40). dditionally, by suppressing superoxide anion production, isotretinoin promotes neutrophil accumulation and dysfunction, features central to chronic granulomatous intestinal inflammation and IBD pathophysiology (41). Nonetheless, further research is needed to fully clarify the relationship between isotretinoin and IBD.

Olmesartan, a selective angiotensin II type 1 receptor (AT1R) antagonist and angiotensin II receptor blocker (ARB), is commonly prescribed for primary hypertension. Recent evidence has established a definitive link between olmesartan and olmesartan-associated sprue-like enteropathy (OAE), a distinct and severe gastrointestinal disorder (42). OAE presents with subtle symptoms that closely mimic Crohn’s disease (CD) in both clinical features and histopathology. A recent study found that among patients with confirmed CD adhering strictly to a gluten-free diet, ARB treatment was significantly associated with persistent or worsening intestinal villous atrophy and elevated clinical symptoms (43). Given these clinical similarities, the immunological mechanisms driving olmesartan-induced intestinal injury may overlap with those underlying IBD. One plausible hypothesis is that olmesartan disrupts intestinal immune balance by attenuating TGF-β signaling, potentially exacerbating pre-existing HLA-DQ2/DQ8–mediated T-cell inflammation in IBD patients and impairing mucosal repair.

Our study also detected positive disproportionality signals in FAERS for teduglutide and ozanimod in association with IBD. However, this correlation requires cautious interpretation. Teduglutide, a recombinant human glucagon-like peptide-2 (GLP-2) analog, is primarily used to treat short bowel syndrome (SBS). Patients with SBS often undergo extensive intestinal resections, leading to structural and functional alterations in the gastrointestinal tract (7). Such resections can create a microenvironment conducive to IBD development or relapse, characterized by gut microbiome dysbiosis, disruption of mucosal immune homeostasis, and compensatory inflammatory responses. Similarly, ozanimod, an oral sphingosine-1-phosphate (S1P) receptor modulator approved for relapsing multiple sclerosis (MS), has been linked to a higher risk of IBD in MS patients compared with non-MS individuals, suggesting shared genetic susceptibility and overlapping immune pathways between MS and IBD (44, 45). Consequently, IBD-related adverse event reports in FAERS associated with teduglutide and ozanimod are likely confounded by underlying disease activity.

Notably, our study is the first to identify positive signals for upadacitinib and risankizumab in relation to IBD, contrasting with the negative findings reported by Zhan et al. (data cutoff: Q4 2023) (46). Given the relatively recent market approval of upadacitinib and risankizumab, early FAERS submissions may have contained insufficient case numbers to detect true signals, potentially yielding false-negative results due to limited statistical power. By extending data extraction to Q2 2025, we increased both drug exposure counts and IBD event reports, thereby enhancing signal detection sensitivity and successfully identifying positive associations for these two agents.

### 4.4 Subgroup analysis

Age-stratified subgroup analysis revealed significant differences in adverse drug reaction signal strength. The reporting disproportionality signal for drug-associated IBD was more pronounced in older adults (≥65 years) than in those under 65. However, this numerical difference may reflect reporting bias, for example, increased concomitant medication use and greater propensity to report adverse events among the elderly. Isotretinoin showed a significantly lower ROR in the older group, likely because it is prescribed more widely in younger populations. Notably, a positive signal emerged between pantoprazole and IBD specifically in older adults. A plausible explanation is that pantoprazole may disrupt the intestinal microbiota barrier by inhibiting gastric acid secretion and directly damage tight junctions in intestinal epithelial cells, thereby affecting the onset and progression of IBD (47). Moreover, advancing age is associated with declining gut microbiota stability and deteriorating barrier function, which may render older adults more susceptible to these adverse effects.

In the sex-stratified analysis, isotretinoin, ozanimod, upadacitinib, and most anti-TNF-α agents exhibited higher RORs in males, whereas doxycycline showed a positive signal only in females. This pattern may stem from fundamental sex-based differences in immune responses to drug exposure. The hypothesis is supported by evidence of testosterone’s proinflammatory potential and sex-specific variations in innate immune signaling pathways, factors that could heighten male susceptibility to certain immune-mediated intestinal toxicities (48). Additionally, gender-related pharmacokinetic differences may contribute to this phenomenon (49).

Encouraging findings also emerged from disease-stratified analysis. The ROR for isotretinoin was higher in ulcerative colitis (UC) patients than in Crohn’s disease (CD) patients, consistent with prior literature suggesting a stronger association with UC (50). Similarly, our results align with previous studies indicating that anti-TNF therapy–induced new-onset IBD adverse reactions occur predominantly as CD: most anti-TNF-α drugs showed higher RORs in CD than in UC patients. Existing research suggests that anti-TNF-α agents may disrupt cytokine balance in individuals carrying genetic susceptibility variants such as *NOD2/CARD15* mutations, thereby triggering CD development (51).

Although age, sex, and disease subtype differences in IBD onset are well documented, spontaneous reporting data cannot disentangle these biological mechanisms from background determinants of case reporting. Therefore, the disproportionate signals identified in subgroup analyses should be interpreted as hypotheses, offering clues for future targeted drug studies that incorporate pathologically confirmed IBD outcomes and adequately control for confounding factors.

### 4.5 Time-to-onset analysis of positive signal drugs

Time-to-event analyses indicate that most drugs exhibit an “early failure” pattern. Importantly, a relatively long median time to onset coexists with a Weibull shape parameter (β) < 1, indicating heterogeneous risk over time: specifically, a higher short-term risk following treatment initiation that gradually declines thereafter, rather than most events clustering within the first year. In contrast, ixekizumab, risankizumab, and teduglutide display random failure characteristics (β ≈ 1), suggesting a sustained risk over time. Our study further shows that the majority of IBD cases occur within one year after medication initiation. Notably, some IBD cases emerge only after the first year of treatment, underscoring the need for continued vigilance for these drugs. Clinicians should therefore tailor individualized monitoring strategies according to specific drug regimens and provide timely intervention and patient education. However, residual confounding cannot be excluded; thus, our findings should be interpreted as suggestive signals. Prospective studies collecting detailed covariate data are needed to validate these observations.

### 4.6 Limitations of this study

This study has several limitations. First, the FAERS database relies on spontaneous reporting, which is subject to variable reporter identity, underreporting, and lack of baseline patient and medication information, potentially compromising signal detection accuracy and reliability. Second, while we examined drugs commonly reported in both JADER and FAERS to maximize randomness and generalizability, significant heterogeneity between the two databases, in case volume, drug exposure patterns, and regional reporting practices, inevitably weakens the statistical power and external validity of cross-database comparisons. Third, limited data availability in JADER and FAERS precludes adjustment for unmeasured confounders, such as pre-existing comorbidities and concomitant medications, which may amplify or distort observed drug–event associations. Finally, FAERS lacks a denominator for actual drug exposure, precluding quantification of absolute risk or confirmation of causal relationships between drugs and adverse events. Future research should focus on elucidating the pathophysiological mechanisms and patient-specific genetic risk factors underlying drug-associated IBD, which is critical for informing clinical decision-making.

## 5 Conclusions

In conclusion, this study examined IBD-related adverse event reports in the FAERS and JADER databases, compiled a list of suspect drugs associated with IBD along with their number of reported cases, and identified specific agents exhibiting significant safety signals. Our analysis revealed that anti-TNF-α inhibitors, anti-leukocyte biologics, conventional immunosuppressants, and antibiotics consistently generated positive disproportionality signals for IBD in both FAERS and JADER. Additionally, isotretinoin and olmesartan showed positive signals specifically in FAERS. Time-to-onset analysis indicated that the median time to IBD onset was within one year for most drug-associated cases. These real-world data may inform precautionary monitoring strategies for IBD and guide future targeted drug safety surveillance. However, given the inherent limitations of spontaneous reporting systems, the findings should be regarded as hypothesis-generating rather than confirmatory of causality. Prospective cohort studies or chart-reviewed case series incorporating detailed clinical data are needed to validate these drug–IBD associations and elucidate their underlying mechanisms.

## Data Availability

The original contributions presented in the study are included in the article/**Supplementary Material**. Further inquiries can be directed to the corresponding author.
